# Acute hemorrhagic cholecystitis with gallbladder rupture and massive intra-abdominal hemorrhage

**DOI:** 10.4322/acr.2020.232

**Published:** 2021-01-20

**Authors:** Zachary Pickell, Krishnan Raghavendran, Maria Westerhoff, Aaron M. Williams

**Affiliations:** 1 University of Michigan, Michigan Medicine, Department of Surgery, Ann Arbor, Michigan, USA

**Keywords:** Cholecystitis, Acute, Gastrointestinal Hemorrhage, General Surgery, Biliary Tract Surgical Procedures

## Abstract

Acute hemorrhagic cholecystitis is a rare, life-threatening condition that can be further complicated by perforation of the gallbladder. We describe a patient with clinical and radiologic findings of acute cholecystitis with a gallbladder rupture and massive intra-abdominal bleeding. Our patient is a 67-year-old male who presented with an ischemic stroke and was treated with early tissue plasminogen activator. His hospital course was complicated by a fall requiring posterior spinal fusion surgery. He recovered well, but several days later developed subxiphoid and right upper quadrant pain and an episode of hemobilia and melena. A computed tomography scan revealed an inflamed, distended gallbladder with indistinct margins and a large hematoma in the gallbladder fossa extending to the right paracolic gutter. The patient also developed hemodynamic instability concerning for hemorrhagic shock. He underwent an emergent laparoscopic converted to open subtotal fenestrating cholecystectomy with abdominal washout for management of his acute hemorrhagic cholecystitis with massive intra-abdominal hemorrhage. Prompt recognition of this lethal condition in high-risk patients is crucial for optimizing patient care.

## INTRODUCTION

Hemorrhagic cholecystitis is a rare, but life-threatening complication of acute cholecystitis.^1^ ^,^ ^2^ Clinical presentation can widely vary, including right upper quadrant pain, nausea, and vomiting.^3^ However, it can also include hematemesis, melena, hemobilia, or even symptoms of biliary obstruction and jaundice. Duration of symptoms can also vary. In severe cases, patients may present with hemoperitoneum and in life-threatening hemorrhagic shock.^4^ There are a number of mechanisms that may contribute to this complication, including trauma, underlying bleeding diathesis, and antiplatelet or anticoagulant use. Although ultrasound is the mainstay in biliary pathology, its use may be limited in hemorrhagic cholecystitis. Computed tomography (CT) can help make an early diagnosis.^5^ We present this case to highlight the importance of early diagnosis and management of this rare, but life-threatening complication of acute cholecystitis.

## CASE REPORT

A 67-year-old man with a past medical history significant for coronary artery disease, atrial fibrillation not on anticoagulation, congestive heart failure (ejection fraction of 20%), prior stroke, and chronic kidney disease (stage 4) presented with stroke symptoms. After presentation, he was treated with tissue plasminogen activator (tPA) and subsequently recovered. Several days later, the patient had an inpatient fall resulting in multiple cervical fractures requiring posterior spinal fusion. After recovering over several days, the patient developed substernal and subxiphoid pain along with nausea. Given his cardiac and stroke history, he was worked up extensively, yet this was unrevealing. After two days, his pain continued and began radiating to his right subcostal region and he continued having nausea. He also reported having new-onset dark melanotic stools within the last 24 hours. Upon further discussion with the patient, he reported having chronic intermittent right upper quadrant, pain, nausea, and vomiting and occasional fevers and chills associated with the episodes.

Given his persistent acute substernal and subxiphoid pain radiating to the right subcostal region and his new onset melanotic stools, laboratory analysis was performed which revealed a leukocytosis to 16,000 (reference range [RR]; 4.0-10.0K/uL) and a hemoglobin which decreased from 14 g/dL to 7.5 (RR; 13.5-17.0 g/dL) compared to the day prior. He had a partial thromboplastin time (PTT) of 25.3 seconds (RR; 22.0-29.0 seconds) and prothrombin time (PT) of 10.8 seconds (RR; 9.2-12.0 seconds) and an international normalize ratio (INR) of 1.1. His platelets were noted to be 63 K/uL (RR; 150-400K/uL) in the setting of his chronic kidney disease (stage IV). During this time, the patient had also been on prophylactic-dose subcutaneous heparin to minimize risk of deep vein thrombosis.

**Figure 1:**
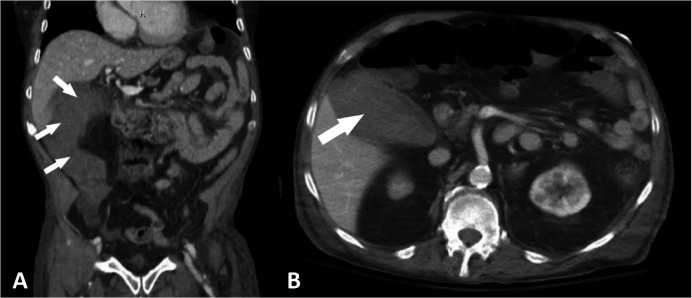
CT abdomen/pelvis demonstrating acute hemorrhagic cholecystitis with perforated/ruptured gallbladder with large heterogenous fluid collection fluid (arrows). Coronal (**A**) and axial (**B**) images.

A computed tomography (CT) scan was performed revealing acute cholecystitis with gallbladder perforation with a right paracolic gutter fluid collection measuring 6.6 x 4.4 x 12 cm in size (Figure 1). Shortly after obtaining the CT scan, the patient started developing hypotension with a systolic blood pressure in the 90-100s mm Hg along with tachycardia to the 100-120s.

**Figure 2:**
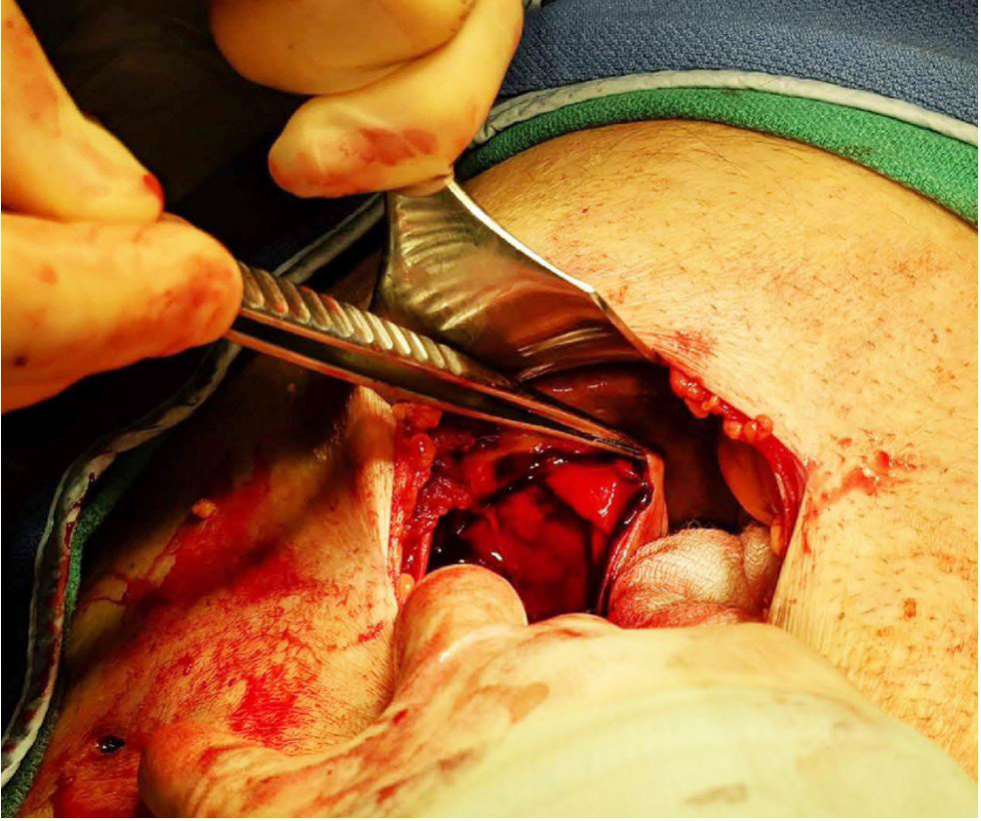
Intraoperative photograph after conversion to open cholecystectomy demonstrating perforation and near complete rupture of gallbladder.

Given the concerning clinical picture, the acute care surgical team was consulted for immediate intervention. Broad-spectrum antibiotics were initiated, and the patient underwent large bore intravenous access, as well as blood product resuscitation. The patient was taken to the operating room for an initial attempt at laparoscopic exploration after sustained improvement in his hemodynamics. Initial laparoscopy revealed a significant amount of blood in the right upper quadrant but did not clearly reveal the gallbladder. Subsequently, the patient underwent conversion to an open subcostal incision. One liter of fresh blood was noted near the gallbladder fossa, which was irrigated and removed. Upon closer evaluation, the gallbladder was noted to be completely ruptured (>75% of gallbladder wall freely open and exposed) with multiple sites of intra-gallbladder active mucosal arterial and venous hemorrhage (Figure 2). This was controlled with a combination of suture ligation and bovie electrocautery.

**Figure 3:**
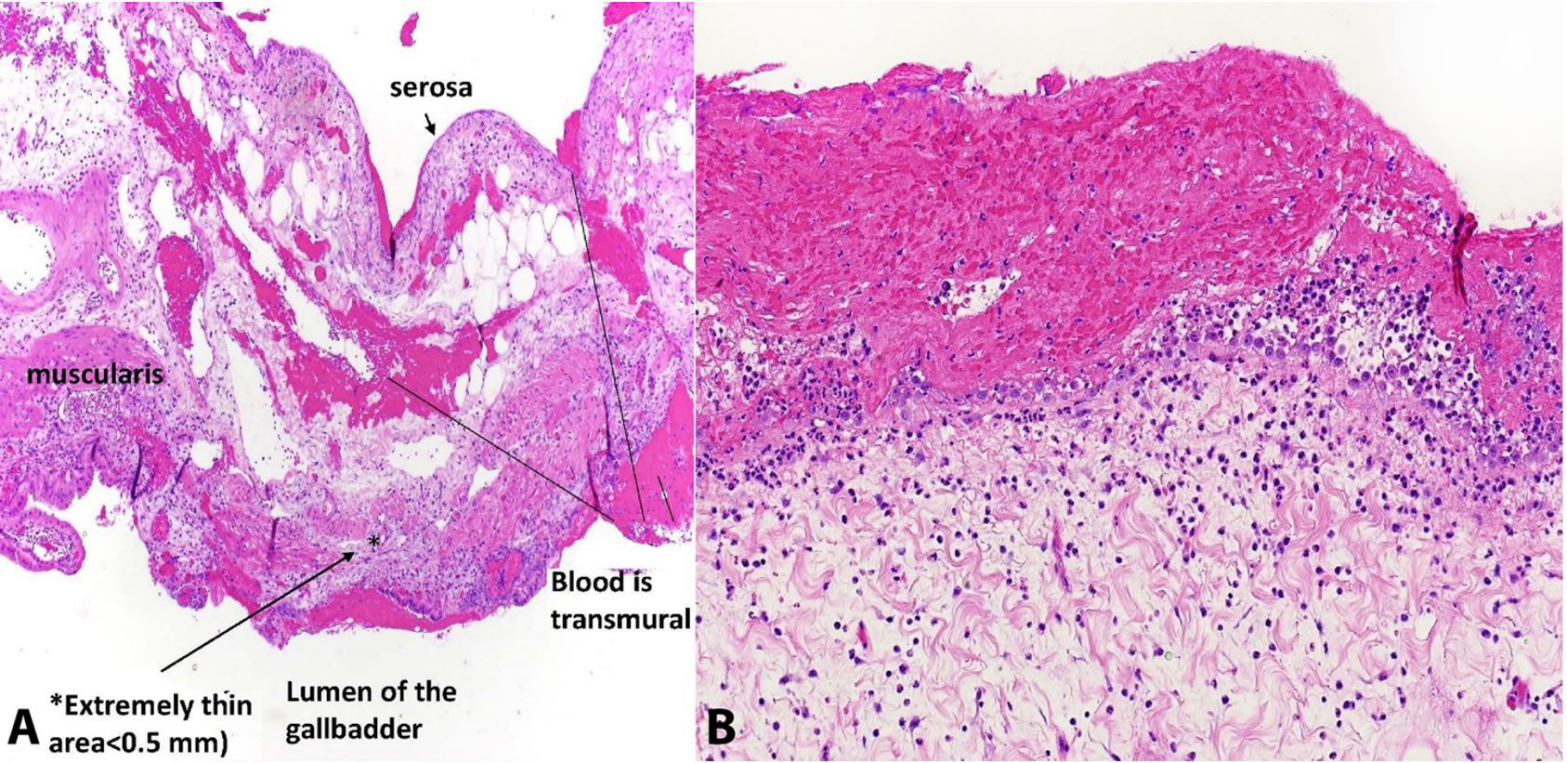
**A –** Photomicrographs of the gall bladder demonstrating acute on chronic hemorrhagic cholecystitis with adherent clotting and blood. In **B –** note fresh blood and peritonitis (not organizing serosal adhesions as would be expected in long term chronic process).

After the bleeding was controlled, a subtotal fenestrating cholecystectomy was performed with a cystic duct orifice purse-string suture and the remaining mucosa underwent electrocautery to minimize risk of fistula. A drain was also placed in the right upper quadrant. The incision was closed in the standard fashion and the patient was transferred to the intensive care unit to recover. Overall, the patient tolerated the operation well and required three units of packed red blood cells, one unit of platelets, one of fresh frozen plasma, and one dose of desmopressin. His lactate and hemoglobin were stable at the end of the case. The patient’s final pathology revealed severe acute on chronic hemorrhagic cholecystitis with evidence of perforation/rupture with focal sites of mucosal bleeding/clot (Figure 3).

The patient recovered well but was noted to have bilious output out of his drain with an elevated drain bilirubin (16 g/dL) several days after surgery. Gastroenterology was consulted, and he underwent an endoscopic retrograde cholangiopancreatography (ERCP), which revealed a biliary leak at the cystic duct. The patient underwent stent placement into his common bile duct for leak management. Three weeks later, the patient was discharged to a rehabilitation facility and was eventually discharged home. His biliary stent was later removed and demonstrated resolution of the leak and his drain was subsequently removed. Nine months later, the patient suffered an unrelated cardiac event with worsening congestive heart failure and passed away.

## DISCUSSION

Acute hemorrhagic cholecystitis is rare and often life threatening.^6^ Because perforation of the gallbladder and subsequent hemorrhage is a rare complication associated with acute cholecystitis, it is important to remain aware of this possible complication. Few studies have previously reported on free rupture of the gallbladder with massive intra-abdominal bleeding.^4^ ^,^ ^7^ ^,^ ^8^ The reported cases focus primarily on increased risk of bleeding in patients with patients on anticoagulants or those who have bleeding diathesis.

Our patient had a history of chronic intermittent right upper quadrant abdominal pain, nausea, and vomiting with the occasional associated fevers and chills. We suspect this is consistent with history of chronic cholecystitis. When he presented to the hospital with a stroke, he received early tPA in the emergency department, which we suspect put him at risk for bleeding from his gallbladder. Combined with his preexisting kidney disease and platelet dysfunction and him receiving prophylactic-dose heparin, this may have placed this patient at a further increased risk for bleeding. This is similar for other cases where patients were taking anticoagulants and presented with hemorrhagic cholecystitis.^4^ ^,^ ^9^ ^-^ ^11^ The patient later presented with substernal and subxiphoid pain that was worked up extensively given his cardiac history. This was likely related to early onset of acute on chronic cholecystitis, which was delayed in diagnosing. This was probably secondary to minimal oral intake after his stroke and for frequent testing and eventual surgery for his spine. After several days, he later presented with signs and symptoms of bleeding along with laboratory and imaging confirmation. We believe the intra-peritoneal gallbladder rupture was secondary to the severe acute on chronic non-cholelithiasic cholecystitis in combination with his intra-gallbladder bleeding. This has been documented in several cases, although transhepatic perforation can occur.^7^ Overall, the present case appears to have only been described in four other cases, to our knowledge.^4^ ^,^ ^9^ ^-^ ^11^ Although the diagnosis is rare, given this patient’s complicated history and risk of bleeding, acute hemorrhagic cholecystitis with gallbladder rupture should remain in the differential diagnosis of patient management.

At present, surgical intervention remains the treatment of choice for acute hemorrhagic cholecystitis with massive intra-abdominal bleeding.^12^ Depending on the source, however, interventional radiology consultation for embolization of a bleeding vessel demonstrating extravasation may also be considered.^13^ ^,^ ^14^ Postoperatively, these patients may also be at high risk for biliary complications, including leak, given the ischemic burden on the biliary system during hemorrhage and shock requiring resuscitation. In these instances, ERCP remains the gold standard for management.^15^ ^-^ ^17^

## CONCLUSION

Patients who present with a several-day history of abdominal pain, nausea, and vomiting with a negative otherwise workup should be evaluated for biliary pathology. Patients with ongoing symptoms for several days and with risk factors for bleeding should be considered for acute hemorrhagic cholecystitis. Rupture of the gallbladder is a rare, life-threatening complication associated with acute hemorrhagic cholecystitis, and can be confirmed with a CT scan. Prompt surgical intervention remains the mainstay for patients with this diagnosis.
